# Supplementary material for: Generation of ultrahigh-brightness pre-bunched beams from a plasma cathode for X-ray free-electron lasers
Source: Nat Commun. 2022 Jun 11;13:3364. doi: 10.1038/s41467-022-30806-6 (PMC9188572; doi:10.1038/s41467-022-30806-6)
Supplement: Supplementary file 1 — Supplementary Information [file 41467_2022_30806_MOESM1_ESM.pdf]

Supplementary Information

1 Supplementary Note 1: Phase velocity of the wake tail in a modulated density ramp

We discuss the modulation of the phase velocity v_ϕ of the wake tail in a density modulated plasma ramp in this section.

1.1 Modulation of v_ϕ in a pre-modulated plasma and a plasma modulated by two counter-propagating laser pulses

When the two counter-propagating laser pulses overlap, the plasma ions move little while the electron density reaches an equilibrium (i.e., a spatial modulated density distribution) where the charge separation force and the ponderomotive force are in balance. When the driver propagates through this electron density modulated region (three pulses, the driver and the two laser pulses, are overlapped), the ponderomotive force of the superimposed lasers plays a similar role on the plasma electron response as the charge separation force of the modulated ion density in a pre-modulated plasma case. Thus, the behavior of the phase velocity in a pre-modulated plasma (modulated electrons and ions) is similar to that when only the electron density is modulated by two counter-propagating lasers. This can be seen by linearizing the fluid equations and Gauss's law in the one-dimensional (1D) linear plasma wakefield regime

$$
\begin{aligned}
&\frac{\partial v_{\mathrm{e1}}}{\partial t} = -\frac{e}{m_{\mathrm{e}}} E_{z1} + F_{\mathrm{p}} \\
&\frac{\partial n_{\mathrm{e1}}}{\partial t} + n_{\mathrm{e0}} \frac{\partial}{\partial z} v_{\mathrm{e1}} = 0 \\
&\frac{\partial}{\partial z} E_{z1} = -4\pi e(n_{\mathrm{e0}} + n_{\mathrm{e1}} - n_{\mathrm{i0}}) - 4\pi e n_{\mathrm{b}}
\end{aligned}
\tag{1}
$$

where m_e and e are the electron mass and charge, n_e0 and n_i0 are the background density of plasma electrons and ions, n_b is the density of charged beam driver, n_e1 is the perturbed plasma electron density, v_e1 is the velocity of plasma electron, E_{z1} is the electric field of the plasma, and F_p is the ponderomotive force when the two counter-propagating laser pulses overlap. For a pre-modulated plasma with $n_\mathrm{e0} = n_\mathrm{i0} = n_\mathrm{p0} + \delta\bar{n}\mathrm{sin}(k_\mathrm{m}z)$ and the ponderomotive force $F_\mathrm{p} = 0$, the linearized equation of the perturbed plasma electron density is

$$\frac{\partial^2 n_\mathrm{e1}}{\partial t^2} + \omega_\mathrm{p0}^2 \left[1 + \delta\hat{n}\mathrm{sin}(k_\mathrm{m}z)\right] n_{e1} = -\omega_\mathrm{p0}^2 \left[1 + \delta\hat{n}\mathrm{sin}(k_\mathrm{m}z)\right] n_\mathrm{b} \tag{2}$$

where $\delta\hat{n} = \delta\bar{n}/n_\mathrm{p0}$ and $\omega_\mathrm{p0}^2 = \frac{4\pi n_\mathrm{p0} e^2}{m_\mathrm{e}}$.

For an initially uniform plasma ($n_\mathrm{e0} = n_\mathrm{i0} = n_\mathrm{p0}$), two counter-propagating lasers first produce a spatial sinusoidal electron density modulation and the 0th-order equations are

$$\begin{aligned} 0 &= -\frac{e}{m_\mathrm{e}} E_{z0} + F_\mathrm{p} \\ \frac{\partial}{\partial z} E_{z0} &= -4\pi e (n_\mathrm{e0} - n_\mathrm{i0}) \end{aligned} \tag{3}$$

It shows the "new" n_e0 is $n_\mathrm{e0} = n_\mathrm{p0} - \frac{m_\mathrm{e}}{4\pi e^2}\frac{\partial}{\partial z}F_\mathrm{p}$. If an electron beam driver excites a plasma wake, then the 1st-order equations are

$$\begin{aligned} \frac{\partial v_\mathrm{e1}}{\partial t} &= -\frac{e}{m_\mathrm{e}} E_{z1} \\ \frac{\partial n_\mathrm{e1}}{\partial t} &+ \left[n_\mathrm{p0} - \frac{m_\mathrm{e}}{4\pi e^2}\frac{\partial}{\partial z}F_\mathrm{p}\right] \frac{\partial}{\partial z} v_\mathrm{e1} = 0 \\ \frac{\partial}{\partial z} E_{z1} &= -4\pi e n_\mathrm{e1} - 4\pi e n_\mathrm{b} \end{aligned} \tag{4}$$

Thus, the perturbed electron density satisfies the same equation as in the pre-modulated plasma case

$$\frac{\partial^2 n_\mathrm{e1}}{\partial t^2} + \omega_\mathrm{p0}^2 \left[1 + \delta\hat{n}\mathrm{sin}(k_\mathrm{m}z)\right] n_\mathrm{e1} = -\omega_\mathrm{p0}^2 \left[1 + \delta\hat{n}\mathrm{sin}(k_\mathrm{m}z)\right] n_\mathrm{b} \tag{5}$$

where $-\frac{m_\mathrm{e}}{4\pi e^2}\frac{\partial}{\partial z}F_\mathrm{p} = \delta\bar{n}\mathrm{sin}(k_\mathrm{m}z) = n_\mathrm{p0}\delta\hat{n}\mathrm{sin}(k_\mathrm{m}z)$ is used.

In order to confirm that the phase velocity of the wake is modulated in both cases, we show 1D simulation results in a linear plasma wakefield regime in Fig. S1. Two cases are considered: one is with a pre-modulated plasma ($\delta\bar{n} = 0.002 n_\mathrm{p0}, k_\mathrm{m} =$

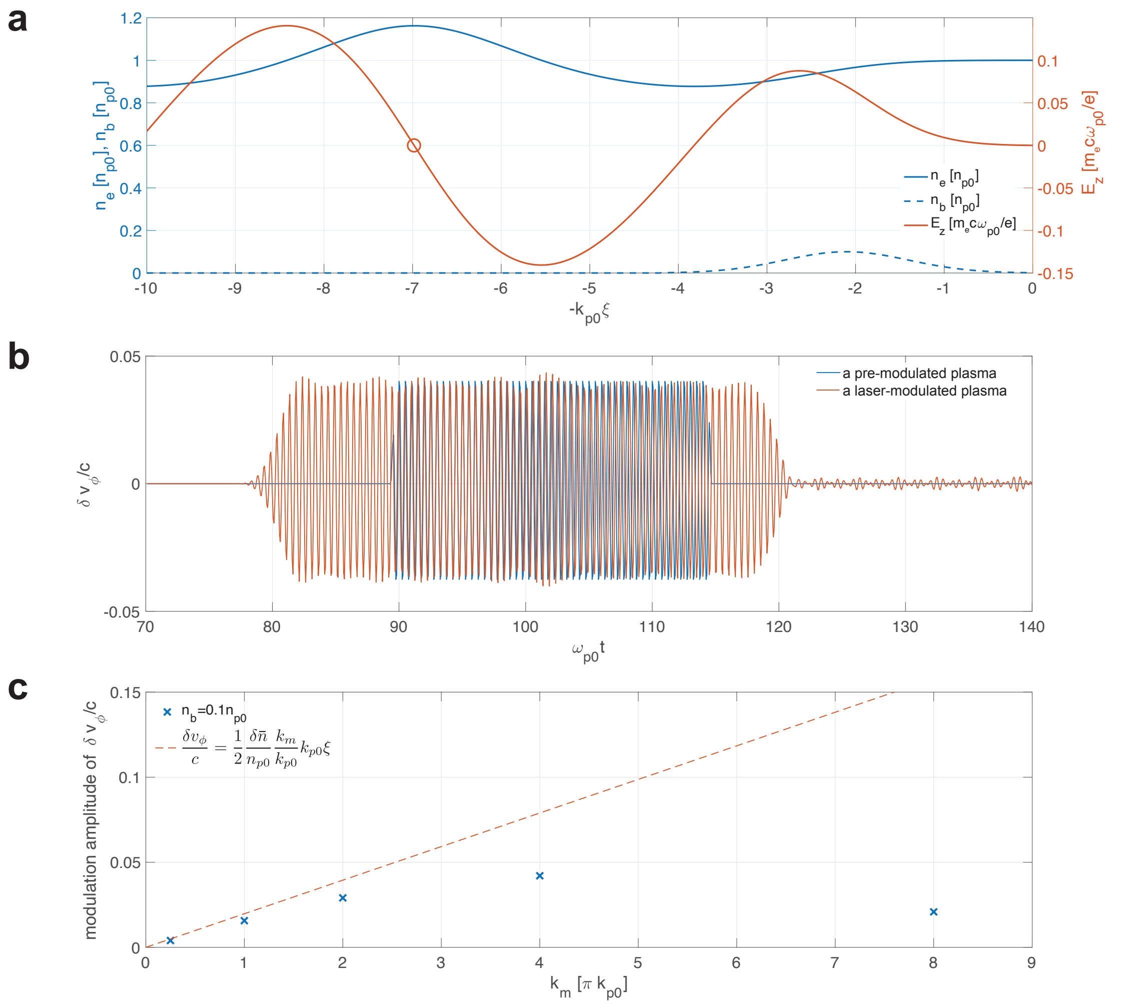

Figure S1: The modulation of the phase velocity in 1D linear plasma wakefield regime. **a** The density of the electron beam driver, the plasma electron and the longitudinal electric field E_z. **b** The variation of the phase velocity of the position where $E_z = 0$ (red circle in a). **c** The comparison of the phase velocity modulation amplitude from simulations and the formula for the pre-modulated plasma case. Parameters: $n_\mathrm{b} = 0.1 n_\mathrm{p0}, k_\mathrm{p0}\sigma_z = 0.7, k_\mathrm{p0} z_\mathrm{i} = -45$; the lasers have the same profile as in Fig. 2 of main text. Note that the density modulation amplitude ($\delta\bar{n} = 0.002 n_\mathrm{p0}$) in the pre-modulated case is set as equal to the expected value generated by two laser pulses.

$4\pi k_{\rm p0}$) and the other uses two counter-propagating lasers ($a_{\rm L0} = 0.005, k_{\rm L} = 2\pi k_{\rm p0}$) to modulate the electrons only. Fig. S1 clearly shows that the phase velocity defined at the location one wave period behind the driver (red circle in Fig. S1a) is modulated with similar period ($0.5k_{\rm p0}^{-1}$) and amplitude ($0.04c$) in both cases. The same idea holds in multi-dimensions although the dynamics is more complicated.

1.2 Modulation of v_ϕ when the density modulation wavelength is shorter than plasma wake wavelength

When the modulation wavelength is much longer than the plasma wake wavelength, the phase velocity in a density modulated ramp is

$$\frac{v_\phi}{c} \approx 1 + \frac{-g + \delta\hat{n}\hat{k}_{\rm m}\cos(\hat{k}_{\rm m}\hat{z})}{2}\hat{\xi} \tag{6}$$

where $\hat{\xi} = \omega_{\rm p0}t - k_{\rm p0}z$ is the normalized position inside the wake, $v_{\rm d}/c$ is assumed to be unity, and $g \equiv \frac{\Delta n/n_{\rm p0}}{k_{\rm p0}L}$ is the normalized density gradient. Normalized units are used to simplify the form where $\delta\hat{n} = \delta\bar{n}/n_{\rm p0}$, $\hat{k}_{\rm m} = k_{\rm m}/k_{\rm p0}$ and $\hat{z} = k_{\rm p0}z$.

In this work, we consider parameters where the modulation wavelength is much shorter than the plasma wake wavelength. In this case, the expression for v_ϕ is not strictly valid. As shown in Fig. S1, the modulation amplitude of v_ϕ is smaller than the theoretical prediction (Eq. 6) when $k_{\rm m} = 4\pi k_{\rm p0}$, $\delta v_\phi \approx c\frac{1}{2}\frac{\delta\bar{n}}{n_{\rm p0}}\frac{k_{\rm m}}{k_{\rm p0}} \times 2\pi \approx 0.08c$. This is because the excursion of the plasma electrons during their oscillations ($0.16k_{\rm p0}^{-1}$ for case $n_{\rm b} = 0.1n_{\rm p0}$) is comparable to the modulation wavelength ($0.5k_{\rm p0}^{-1}$), thus they experience a varying ion density which is equivalent to a reduced density modulation. When the electrons oscillation amplitude is much less than the modulation wavelength, the phase velocity is modulated with an amplitude close to Eq. 6 as shown in Fig. S1c.

We can understand this by considering the oscillation equation of the plasma electron. Consider a plasma electron whose position is $z = z_0 + Z(z_0)$, where z_0 and $Z(z_0)$ are the equilibrium position and displacement from its equilibrium position. Assuming there is no trajectory crossing, the electric field at the electron in 1D geometry is $E_z = 4\pi e\int_{z_0}^{z_0+Z}{\rm d}z n_{\rm p}(z)$, thus the equation of motion for the electron is

$$\frac{{\rm d}^2 Z}{{\rm d}t^2} = -\omega_{\rm p0}^2\int_{z_0}^{z_0+Z}{\rm d}z\frac{n_{\rm p}(z)}{n_{\rm p0}} \tag{7}$$

For a pre-modulated plasma with $n_\mathrm{p}(z) = n_{\mathrm{p0}} + \delta\bar{n}\sin(k_\mathrm{m}z)$,

$$\frac{\mathrm{d}^2 Z}{\mathrm{d}t^2} = -\omega_{\mathrm{p0}}^2 \left[Z + \delta\hat{n}\frac{-\cos[k_\mathrm{m}(z_0 + Z)] + \cos(k_\mathrm{m}z_0)}{k_\mathrm{m}} \right] \tag{8}$$

To our knowledge, there is no analytical solution for the above differential equation. In the $k_\mathrm{m}Z \ll 1$ limit, an approximate solution is $Z = Z_\mathrm{i}\sin\left[\omega_{\mathrm{p0}}\sqrt{1+\delta\hat{n}\sin(k_\mathrm{m}z_0)}t\right]$ where Z_i is the initial displacement at $t = 0$. We solve this oscillation equation numerically and summarize the results in Fig. S2. When $k_\mathrm{m}Z \ll 1$, the particle oscillates with the local plasma frequency which deviates from ω_{p0}; as $k_\mathrm{m}Z$ becomes larger, this deviation decreases because the particle experiences a varying ion density.

The physics becomes much more complicated in the multi-dimensional nonlinear plasma wakefield regime. The axial and radial displacement of the electrons can be much larger than the density modulation wavelength. We thus rely on simulations to study the modulation of the phase velocity in the 3D blowout regime. The results are shown in Fig. 1b of main text where the modulation amplitude is also reduced.

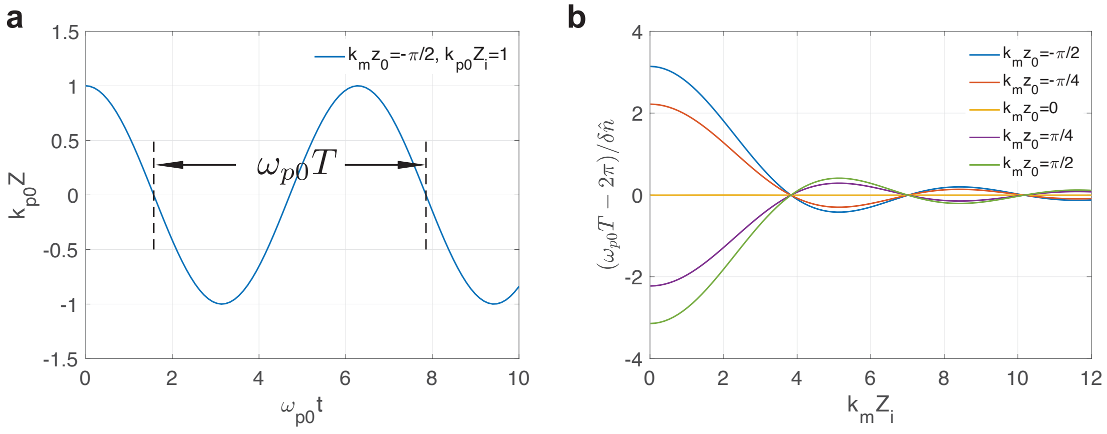

Figure S2: The reduction of the modulation amplitude of v_ϕ when $k_\mathrm{m} \gg k_{\mathrm{p0}}$. **a** The oscillation of a single electron. **b** The deviation of the oscillation period (defined in **a**) for particles with different equilibrium positions and initial displacements. Parameters: $k_\mathrm{m} = 4\pi k_{\mathrm{p0}}, \delta\hat{n} = 0.002$.

2 Supplementary Note 2: Plasma density modulation excited by two counter-propagating lasers

We analyze the density perturbation driven by the ponderomotive force of two counter-propagating laser pulses of the same frequency. For simplicity, we consider a plasma with uniform density n_{p0} centered about $z = 0$. Two finite length laser pulses propagate in opposite directions. Assuming the lasers are polarized along x-direction, propagate in the z-direction, and they have the same envelop, the vector potential of the two lasers is

$$A_{\mathrm{L}}\hat{x} = -\frac{m_{\mathrm{e}}c}{e}a_{\mathrm{L0}}\left[f(z_0 + k_{\mathrm{L}}z - \omega_{\mathrm{L}}t)\cos(k_{\mathrm{L}}z - \omega_{\mathrm{L}}t) - f(z_0 - k_{\mathrm{L}}z - \omega_{\mathrm{L}}t)\cos(k_{\mathrm{L}}z + \omega_{\mathrm{L}}t)\right] \tag{9}$$

and the electromagnetic fields are $E_{\mathrm{L}}\hat{x} = -\frac{\partial A_{\mathrm{L}}}{\partial t}\hat{x}, B_{\mathrm{L}}\hat{y} = \frac{\partial A_{\mathrm{L}}}{\partial z}\hat{y}$.

The transverse momentum of the plasma electrons and ions can be obtained through the conservation of transverse canonical momentum, i.e.,

$$\frac{v_x}{c} = \frac{p_x}{m_{\mathrm{e}}c} = \frac{eA_{\mathrm{L}}}{m_{\mathrm{e}}c^2}, \frac{v_{x\mathrm{i}}}{c} = \frac{p_{x\mathrm{i}}}{m_{\mathrm{i}}c} = \frac{eA_{\mathrm{L}}}{m_{\mathrm{i}}c^2} \tag{10}$$

where the motion is assumed to be non-relativistic, i.e., $\frac{eA_{\mathrm{L}}}{m_{\mathrm{e}}c^2} \equiv a_{\mathrm{L}} < 1$, and v_x and $v_{x\mathrm{i}}$ are the transverse velocities of the plasma electrons and ions, m_{e} and m_{i} are the mass of electron and ion.

We use Euler's equations for a cold plasma, the continuity equation, and the Poisson's equation where only the leading terms are kept,

$$\frac{\partial v_z}{\partial t} = -\frac{e}{m_{\mathrm{e}}}(v_x B_{\mathrm{L}} + E_z), \frac{\partial v_{z\mathrm{i}}}{\partial t} = \frac{e}{m_{\mathrm{i}}}(v_{x\mathrm{i}} B_{\mathrm{L}} + E_z) \tag{11}$$

$$\frac{\partial \delta n}{\partial t} + n_{\mathrm{p0}}\frac{\partial v_z}{\partial z} = 0, \frac{\partial \delta n_{\mathrm{i}}}{\partial t} + n_{\mathrm{p0}}\frac{\partial v_{z\mathrm{i}}}{\partial z} = 0 \tag{12}$$

$$\frac{\partial E_z}{\partial z} = 4\pi e(-\delta n + \delta n_{\mathrm{i}}) \tag{13}$$

From these it follows that ,

$$\frac{\partial^2 \delta n}{\partial t^2} + \omega_{\mathrm{p0}}^2 \delta n = \frac{c^2}{2} n_{\mathrm{p0}} \frac{\partial a_{\mathrm{L}}^2}{\partial z^2} + \omega_{\mathrm{p0}}^2 \delta n_{\mathrm{i}} \tag{14}$$

$$\frac{\partial^2 \delta n_{\mathrm{i}}}{\partial t^2} + \omega_{\mathrm{pi}}^2 \delta n_{\mathrm{i}} = \frac{c^2}{2} \frac{m_{\mathrm{e}}^2}{m_{\mathrm{i}}^2} n_{\mathrm{p0}} \frac{\partial a_{\mathrm{L}}^2}{\partial z^2} + \omega_{\mathrm{pi}}^2 \delta n \tag{15}$$

where $\omega_{p0} = \sqrt{\frac{4\pi e^2 n_{p0}}{m_e}}$ and $\omega_{pi} = \sqrt{\frac{4\pi e^2 n_{p0}}{m_i}}$ are the electron and ion plasma frequencies respectively. The ponderomotive force in Eq. 15 on the ions is smaller than that on the electrons by $\left(\frac{m_e}{m_i}\right)^2$ and can thus be neglected. The ions thus only respond to the space charge force from the plasma electron bunching/debunching. In the limit of relatively short lasers or large mass ratios, i.e., $\delta n_i \approx 0$ and in many cases the motion of the ions can be neglected.

The envelope $f(z)$ is assumed to be slow varying function compared with the laser frequency, thus

$$\frac{\partial^2(\delta\hat{n})}{\partial\hat{t}^2} + \delta\hat{n} \approx (a_L\hat{\omega}_L)^2\cos(2\hat{k}_L\hat{z})\left(2f(z_0+\xi_1)f(z_0-\xi_2) - \left[f^2(z_0+\xi_1)+f^2(z_0-\xi_2)\right]\cos(2\hat{\omega}_L\hat{t})\right) - (a_L\hat{\omega}_L)^2\sin(2\hat{k}_L\hat{z})\left[f^2(z_0+\xi_1)-f^2(z_0-\xi_2)\right]\sin(2\hat{\omega}_L\hat{t}) \tag{16}$$

where $\xi_1 = k_L z - \omega_L t$ and $\xi_2 = k_L z + \omega_L t$.

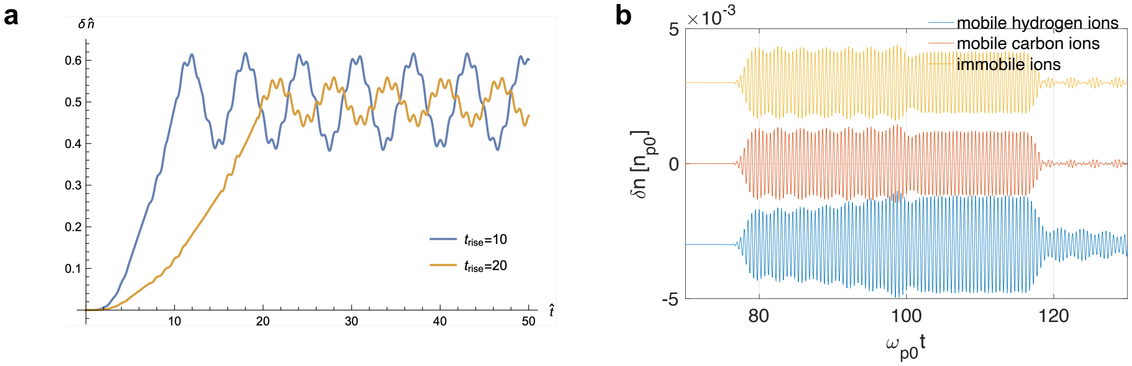

Figure S3: The modulation of plasma electron density excited by two counter-propagating lasers. **a** The evolution of the perturbed plasma density with $t_{rise} = 10$ and $t_{rise} = 20$. **b** The density modulation along the black dashed trajectory in Fig. 5a of main text when $\omega_L = 2\pi\omega_{p0}, a_{L0} = 4\times10^{-3}$. The laser has the same profile as in Fig. 2 of main text. Note the value of density is offset by -0.003, 0 and 0.003, respectively.

To illustrate the response, we consider the position with $z = 0$ and ignore the ion response to obtain,

$$\frac{\partial^2(\delta\hat{n})}{\partial\hat{t}^2} + \delta\hat{n} \approx 2(a_L\hat{\omega}_L)^2 f^2(z_0+\xi_1)\left[1-\cos(2\hat{\omega}_L\hat{t})\right] \approx 2(a_L\hat{\omega}_L)^2 f^2(z_0+\xi_1) \tag{17}$$

where we average over the laser frequency such that the high frequency term of the right hand of the equation (i.e., $2\hat{\omega}_{\rm L}$ term) is neglected. This could be solved for formally using Green's function. However, if $f(z)$ changes slowly on the time scale of plasma period, we can neglect the second derivative term on the left hand side to

$$\delta\hat{n} \approx 2(a_{\rm L}\hat{\omega}_{\rm L})^2 f^2(z_0 + \xi_1) \tag{18}$$

The general solution will have small amplitude oscillations at the natural frequency whose amplitude is determined by how rapidly $f(z)$ rises. We show two numerical examples in Fig. S3a. The envelope function rises linearly from 0 to 1 during $t_{\rm rise}$ and then stays constant. The perturbed density follows $f^2(z)$ in both cases but has a larger amplitude oscillation for $t_{\rm rise} = 10$. The perturbed density at other z has similar behaviors as at $z = 0$.

In Fig. S3b, we present 1D PIC simulations using the particle-in-cell code OSIRIS to compare the density modulation when immobile ions and mobile ions are used for parameters of relevance to the article. We can see the density modulation is slightly deeper with mobile hydrogen ions. When mobile carbon ions are used, the modulation is the same as the case with immobile ions.

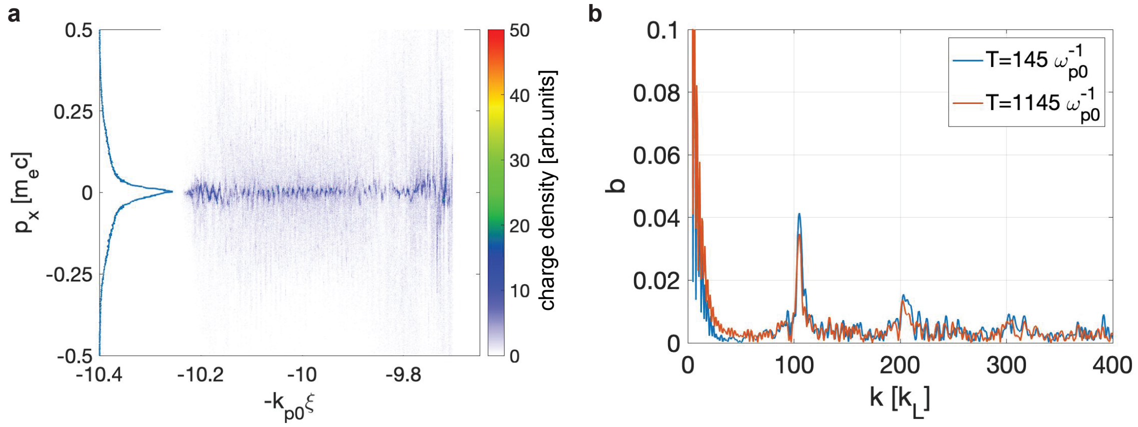

Figure S4: The effect of the betatron motion and the energy spread on the bunched structure. **a** The charge density distribution of the injected electrons in $p_x - \xi$ space at $145\omega_{\rm p0}^{-1}$. The line is the projection of electron with $-k_{\rm p0}\xi \leq -9.85$. **b** The comparison of the bunching factor at the end of PIC simulations and when they are accelerated to 1.08 GeV.

3 Supplementary Note 3: The effect of the betatron motion and the energy spread on the bunched structure

We accelerate the electrons in Fig. 2 of main text to 1.08 GeV by solving the equations of motion where the slippage induced by the energy spread and the betatron motion are included. The external fields are $E_x = \frac{x}{2}\frac{m_e\omega_{p0}^2}{e}$, $E_y = \frac{y}{2}\frac{m_e\omega_{p0}^2}{e}$, $E_z = -2\frac{m_e c\omega_{p0}}{e}$. Their six-dimensional phase space distribution at $145\omega_{p0}^{-1}$ serves as the initial conditions. The bunching factor is shown in Fig. S4b. We can see it changes little compared with the bunching factor at the end of the PIC simulation ($145\omega_{p0}^{-1}$).

4 Supplementary Note 4: Summarization of the parameters for the simulation shown in Fig. 2 of main text

In Supplementary Table 1, we summarize the parameters of the colliding lasers, the driver beam and the injected beam for the simulation shown in Fig. 2 of main text.

Supplementary Table 1 Parameters of Fig. 2 of main text ($n_{p0} = 1.97 \times 10^{19}$ cm^{-3}).

	Parameter	Value	Unit
Lasers	λ_{L0}	1.2	µm
	a_L	0.005	
	w_0	7.2	µm
	$t_{rise}, t_{flat}, t_{fall}$	(40, 280, 40)	fs
Beam driver	E_d	2	GeV
	I_d	34	kA
	σ_r, σ_z	(0.6, 0.84)	µm
Injected beam	I_b	$\sim$ 17	kA
	ϵ_N	$\sim$ 7	nm
	σ_{E_b}	$\sim$ 0.4	MeV

Supplementary Table 1: The physical parameters of the colliding lasers, the driver and the injected beam for the self-consistent simulation shown in the main text.

5 Supplementary Note 5: GENESIS 1.3 simulation results

5.1 Transport and FEL process

The basic design for transporting the beam exiting the plasma to the undulator without significant debunching is shown in Fig. S5. The 1.09 GeV bunched beam with 3.6 nm bunched structure propagates through a matching plasma with density profile $n_{\mathrm{p}}(z) = \frac{n_{\mathrm{p0}}}{[1+(z-z_{\mathrm{m}})/l]^2}$, where $l \approx 47$ µm and the total length of the plasma matching section is $L = 0.1$ m, and z_{m} is the start of the matching section. It then drifts $L_{\mathrm{d}} = 0.1$ m in free space to reach the undulator. For the values of L an L_{d} used here the bunching factor is preserved. The GENESIS 1.3 simulation result is shown in Fig. 4 of main text. Note, external focusing magnets are absent and the natural focusing force from the undulator can be neglected in such a short distance. The spot size of the beam grows by a factor of ~ 2 by the end of the simulation ($z = 0.6$ m).

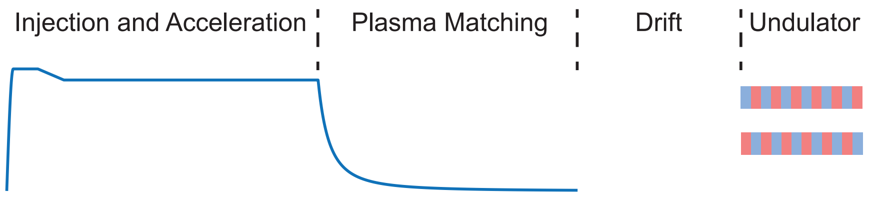

Figure S5: A conceptual plot of the plasma-based accelerator driven XFEL. See the text for the actual distances and sizes of the various physical elements shown above.

5.2 Signal to noise

As shown in Fig. 2e of main text, there is a large current spike at the bunch head and it will produce X-ray radiation through SASE in the undulator. However, the large current beam head has a larger emittance and a larger energy spread than the modulated beam core: $\sim 8\ I_{\mathrm{A}}$ v.s. $\sim I_{\mathrm{A}}$, $\sim 0.06 k_{\mathrm{p0}}^{-1}$ v.s. $\sim 0.006 k_{\mathrm{p0}}^{-1}$ and ~ 16 MeV v.s. ~ 0.4 MeV (see Fig. S6). Thus, the 6D brightness of the head is lower by a factor of ~ 500 than that of the beam core which indicates the head would generate much lower radiation compared with the pre-bunched core in a short undulator. The GENESIS simulation shows a power contrast as 234 GW (beam core) v.s. 8 GW

(beam head) at $z = 0.3$ m. Note the above GENESIS simulation only models the region with a transverse size of 80 microns in each direction, therefore particles that make greater excursions are removed automatically. Furthermore, the current spike may be eliminated when a smaller wake is excited (see case 2 and 3 in Fig. S7) or perhaps when a smoother density ramp is used.

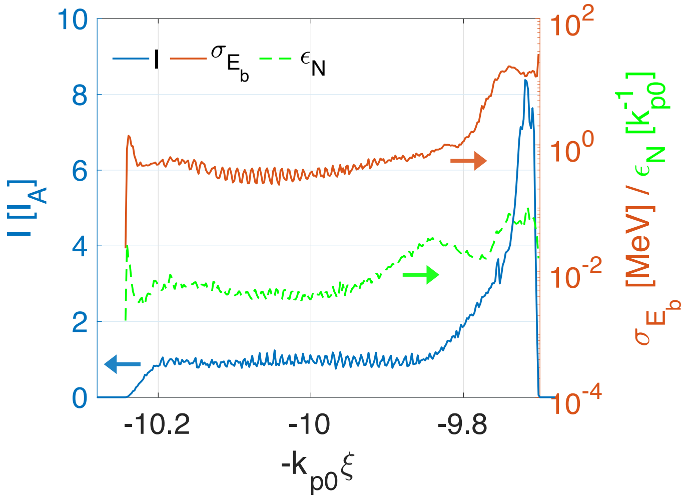

Figure S6: The slice parameters of the injected beam. The blue, green and red lines show the current, emittance and energy spread of the injected beam described in Fig. 2 of main text at $145\omega_{p0}^{-1}$.

6 Supplementary Note 6: Considerations on drivers

In Fig. S7, we show the current profile and the bunching factor of the injected beams for different electron beam driver parameters. The comparison between case 1 and case 4 shows that the current and the bunching factor of the injected beams are not sensitive to the energy, energy spread, and emittance of the driver. The low current case (case 2) and the wide driver case (case 3) produce beams with lower current and larger harmonic number. We also show results from a laser-driven case in Fig. S8 where a pre-bunched beam with harmonic number $h \approx 77$ is produced. There is room for possible improvement for laser drivers. Using a particle beam driver (generated from a conventional accelerator or from a laser plasma wakefield accelerator) or a laser driver have their own advantages and weaknesses. The tightly focused and short beams generated from laser-driven plasma accelerators can be used as drivers

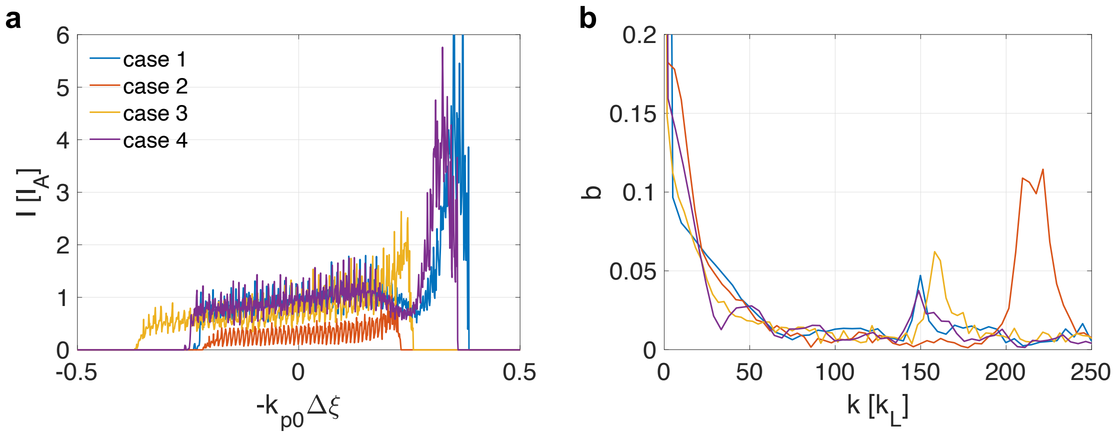

Figure S7: Comparison of the injected bunched beams when different beam drivers are used. **a** The current profile; **b** the bunching factor. To save the computational cost, we use a pre-modulated plasma with $\delta\bar{n} = 10^{-3} n_{\mathrm{p0}}, g = 3 \times 10^{-3}$ and $k_{\mathrm{p0}}\lambda_{\mathrm{m}} = 1$. Case1: reference case, $E_{\mathrm{d}} = 2$ GeV, $\sigma_{E_{\mathrm{d}}} = 0, \epsilon_{\mathrm{N,d}} = 0, I_{\mathrm{d}} = 34$ kA, $k_{\mathrm{p0}}\sigma_z = 0.7, k_{\mathrm{p0}}\sigma_r = 0.5$; case 2: low current driver case, $E_{\mathrm{d}} = 2$ GeV, $\sigma_{E_{\mathrm{d}}} = 0, \epsilon_{N,\mathrm{d}} = 0, I_{\mathrm{d}} = 17$ kA, $k_{\mathrm{p0}}\sigma_z = 0.7, k_{\mathrm{p0}}\sigma_r = 0.5$; case 3: wide driver case, $E_{\mathrm{d}} = 2$ GeV, $\sigma_{E_{\mathrm{d}}} = 0, \epsilon_{\mathrm{N,d}} = 0, I_{\mathrm{d}} = 34$ kA, $k_{\mathrm{p0}}\sigma_z = 0.7, k_{\mathrm{p0}}\sigma_r = 1.25$; case 4: $E_{\mathrm{d}} = 0.5$ GeV, $\sigma_{E_{\mathrm{d}}} = 10$ MeV, $\epsilon_{\mathrm{N,d}} = 1$ μm, $I_{\mathrm{d}} = 34$ kA, $k_{\mathrm{p0}}\sigma_z = 0.7, k_{\mathrm{p0}}\sigma_r = 0.5$.

in a plasma with higher density ($\geq 10^{20}$ cm^{-3}) to produce pre-bunched beams with shorter bunching wavelength and smaller emittance while the use of a laser driver could lead to simpler and more compact design.

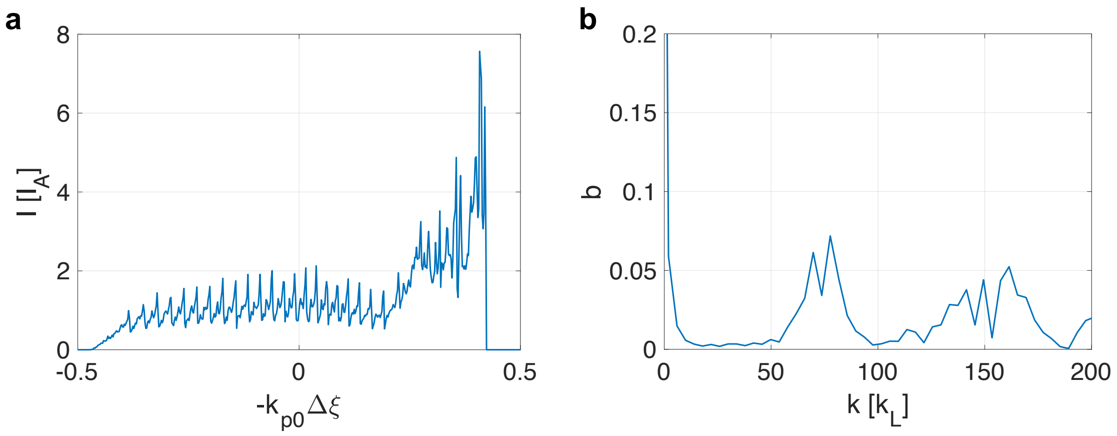

Figure S8: The current profile (**a**) and the bunching factor (**b**) of the injected beam in a laser-driven plasma wake. Parameters: the laser has a spot size $w_0 = 7.6$ µm, and a duration $\tau_{\mathrm{FWHM}} = 28.4$ fs; a pre-modulated plasma downramp with $\delta\bar{n} = 10^{-3}n_{\mathrm{p0}}$, $g = 3 \times 10^{-3}$ and $k_{\mathrm{p0}}\lambda_{\mathrm{m}} = 1$ is used and $n_{\mathrm{p0}} = 7.73 \times 10^{18}$ cm^{-3}.
